# Supplementary material for: Study on the Synthesis of Nano Zinc Oxide Particles under Supercritical Hydrothermal Conditions
Source: Nanomaterials (Basel). 2024 May 12;14(10):844. doi: 10.3390/nano14100844 (PMC11123760; doi:10.3390/nano14100844)
Supplement: Supplementary file 1 [file nanomaterials-14-00844-s001.zip › nanomaterials-2951679-supplementary.pdf]

Supplementary Material

Study on the Synthesis of Nano Zinc Oxide Particles under Supercritical Hydrothermal Conditions

Panpan Sun [1,*], Zhaobin Lv [1] and Chuanjiang Sun [1]

[1] College of Mechanical & Electrical Engineering, Shaanxi University of Science & Technology, Xi'an, Shaanxi, 710021, China; lvzb@sust.edu.cn(Z.L.); 230511036@sust.edu.cn(C.S.);

* Correspondence: panpansunch@sust.edu.cn(P.S.);

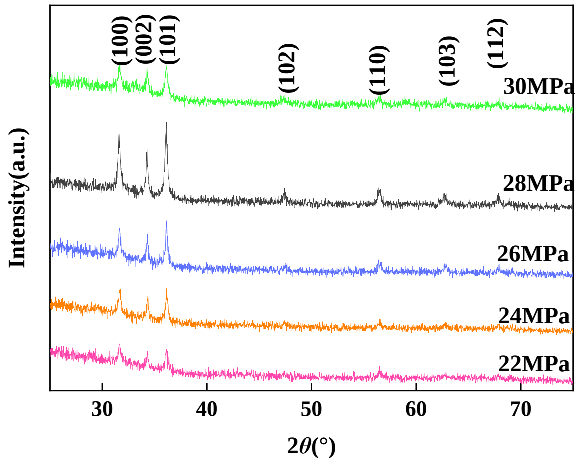

Figure S1. XRD spectra of nano ZnO synthesized under different pressure conditions.

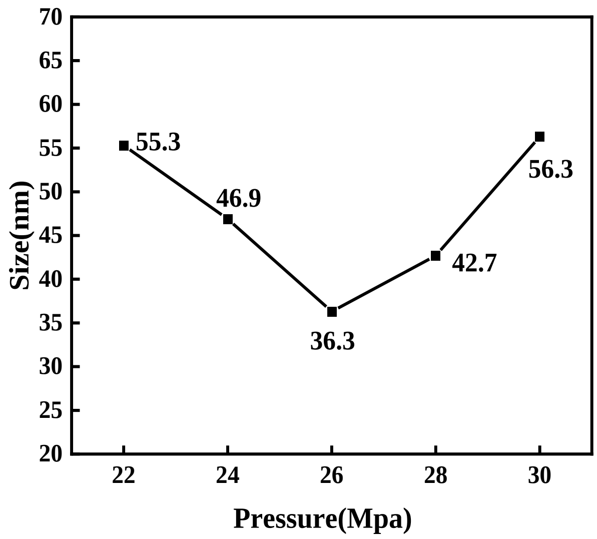

Figure S2. Variation in crystallite size of ZnO synthesized under

22MPa to 30Mpa.

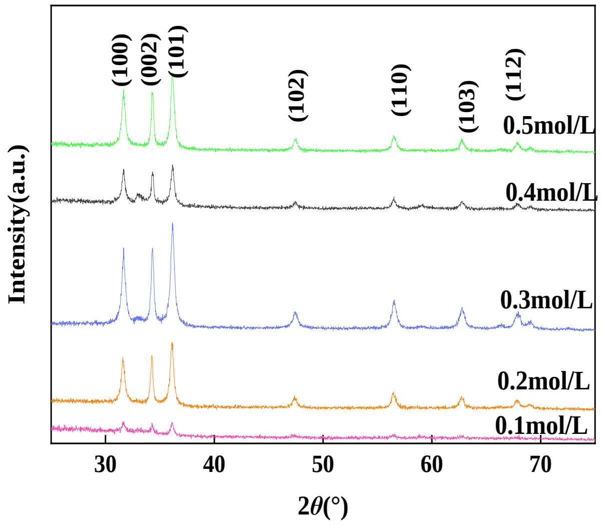

Figure S3. XRD spectra of nano ZnO synthesized under different precursor concentrations.

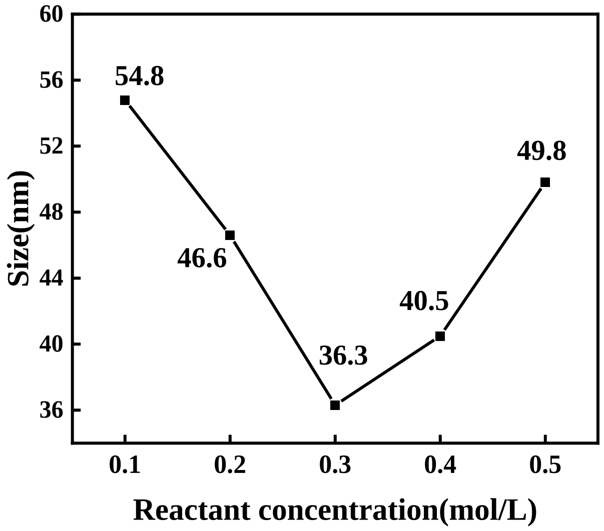

Figure S4. Variation in crystallite size of ZnO synthesized under different precursor concentrations.

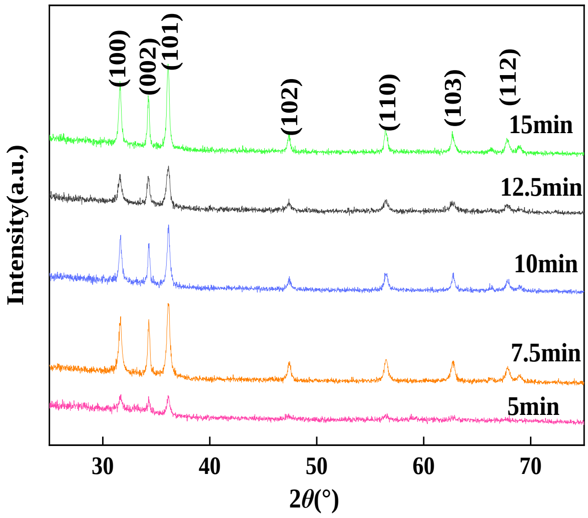

Figure S5. XRD spectra of nano ZnO synthesized under different reaction times.

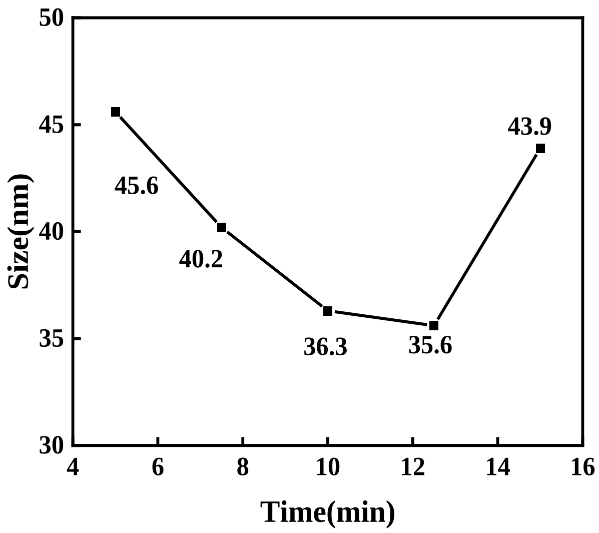

Figure S6. Variation in crystallite size of ZnO synthesized under different reaction times.

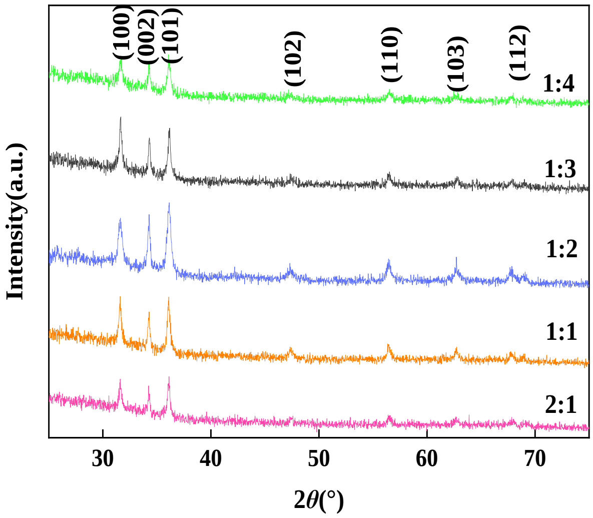

Figure S7. XRD spectra of nano ZnO synthesized under different amounts of ethanol added.

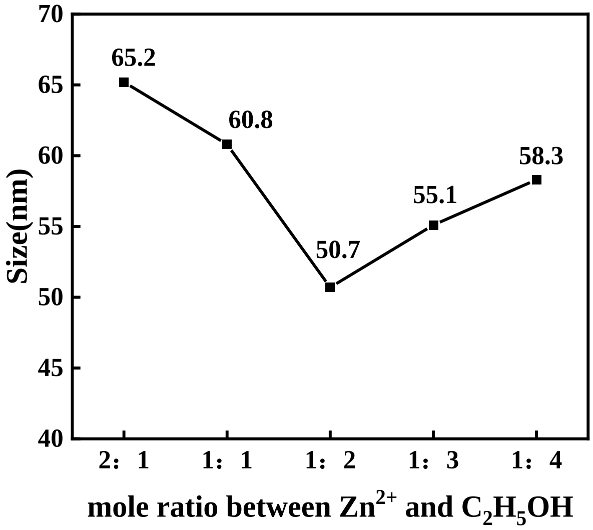

Figure S8. Variation in crystallite size of ZnO synthesized under different amounts of ethanol added.
